# Sample size and classification error for Bayesian change-point models with unlabelled sub-groups and incomplete follow-up

**DOI:** 10.1177/0962280216662298

**Published:** 2016-08-08

**Authors:** Simon R White, Graciela Muniz-Terrera, Fiona E Matthews

**Affiliations:** 1 MRC Biostatistics Unit, Cambridge, UK; 2 School of Clinical Sciences, University of Edinburgh, Edinburgh, UK; 3 Institute of Health and Society, Faculty of Medicine, Newcastle University, Newcastle, UK

**Keywords:** Change-point, change-point regression, broken-stick, sample size, classification, cognitive decline, simulation study, Mini-Mental State Examination

## Abstract

Many medical (and ecological) processes involve the change of shape, whereby one trajectory changes into another trajectory at a specific time point. There has been little investigation into the study design needed to investigate these models. We consider the class of fixed effect change-point models with an underlying shape comprised two joined linear segments, also known as broken-stick models. We extend this model to include two sub-groups with different trajectories at the change-point, a change and no change class, and also include a missingness model to account for individuals with incomplete follow-up. Through a simulation study, we consider the relationship of sample size to the estimates of the underlying shape, the existence of a change-point, and the classification-error of sub-group labels. We use a Bayesian framework to account for the missing labels, and the analysis of each simulation is performed using standard Markov chain Monte Carlo techniques. Our simulation study is inspired by cognitive decline as measured by the Mini-Mental State Examination, where our extended model is appropriate due to the commonly observed mixture of individuals within studies who do or do not exhibit accelerated decline. We find that even for studies of modest size (*n* = 500, with 50 individuals observed past the change-point) in the fixed effect setting, a change-point can be detected and reliably estimated across a range of observation-errors.

## 1 Introduction

When observing a changing outcome over time, using longitudinal data, the process may contain periods in which a marked or distinct change occurs in the underlying shape of the data. The distinct shift from one shape to another is called a change-point.

Change-point models – also known as change-point regression, switching regression,^1^ changing regression,^2^ two-phase regression, segmented regression, broken-stick regression, turning points^3^ or bent-cable regression^4^ – encompass a wide class of problems. They have been fitted to many longitudinal processes such as: modelling distinct changes in the rates of a Poisson process for mining accidents,^5,6^ changes in economic time-series trends,^7^ extremes of climate,^8^ modelling cognitive decline,^9^ effect of calcium supplementation on blood pressure,^10^ CD4 T-cell counts for HIV-infected individuals^11^ and biomarker levels for prostate cancer^12,13^ (see also annotated bibliographies and overviews^14–17^).

In our research setting, which is primarily the study of the longitudinal effects of ageing, individuals experience cognitive decline. However, some individuals experience a period of steep decline, so-called accelerated decline. This naturally leads us to consider change-point models to account for the shift from typical decline to accelerated decline, but fundamental is the concept that not all individuals experience this change. Accelerated decline is a strong precursor of increased mortality and decreasing quality of life. Hence being able to identify sub-groups that are likely to follow different paths is an important area of research, and it is vital to invest in well-designed studies with sufficient statistical power.

### 1.1 Sub-groups

Within the change-point literature, there has been a focus on fitting a common underlying trajectory, with a change-point, to every individual and investigation of several aspects of this trajectory; for example, inferring the time of the change-point^18^ or deriving statistical tests for the existence of a change-point.^19^ However, in many real-world applications, the cohort may be heterogeneous, with individuals following different trajectories.

A key research question is to learn firstly, if there are different classes of individual and secondly, what features identify these individuals. The sub-groups of individuals, namely groups of individuals following different trajectories, are not observed and must be inferred; individuals are unlabelled within the data, hence the term unlabelled sub-groups.

Given a set of classes, there will be uncertainty when inferring the individuals’ labels, and some individuals will be incorrectly labelled, this is classification-error.

### 1.2 Incomplete follow-up

Attrition is a well-known problem in cohort studies^20,21^ and presents a specific challenge when considering change-point models. If the majority of individuals have dropped out of the study before the change-point, the statistical power to detect a change-point and attempts to classify individuals will be severely limited.

To account for attrition in longitudinal studies, we consider incomplete follow-up using a monotone missing assumption, that is when an individual misses a wave they do not return for any future waves; this drop-out mechanism is common to many cohort studies.

Under monotone missingness, we define a sample size metric, specific to the single-change-point model, which we term the expected post-change-point sample size. The expected post-change-point sample size combines the first wave sample size with the real-world problem of attrition in a manner that is intuitive for study designers.

Any discussion of incomplete follow-up must include the missingness mechanism, typically classed as either: missing completely at random (MCAR), missing at random (MAR) or missing not at random (MNAR).^22^

We consider MCAR as a way to include incomplete follow-up in our investigation of classification-error and expected post-change-point sample size, without obscuring these aspects with complex missingness mechanisms, namely investigating how the proportion of random attrition impacts the power to detect a change-point and classification-error in relation to our newly defined sample size metric.

### 1.3 Bayesian framework

Change-point models have been considered using frequentist^23^ and Bayesian^5,24^ approaches. In a Bayesian framework, the extension of the model to incorporate missing data is conceptually simple, though not always computationally possible. The aim of our paper is to investigate change-point models dealing with attrition; this requires a computationally tractable model.

We have two distinct forms of missing data, the unknown sub-group labels and incomplete follow-up. The missing sub-group labels are the motivation for our paper, whereas the incomplete follow-up is essential to the practical application of our results. These forms of missing data are simple to include in a Bayesian change-point model, resulting in a tractable likelihood. Hence, we decided to focus on Bayesian change-point models.

### 1.4 Study design

Study design for change-point models is challenging due to the non-linear nature of the model. Bischoff and Miller^25^ derived frequentist optimal designs to detect the existence of a change-point in the single-path setting. Atherton et al.,^26^ also for the single-path setting but in the Bayesian framework, investigated the optimal location for observation times. There has been little work on optimal design for the so-called multi-path change-point problem, which is the setting of our paper, with repeated observations on multiple individuals.

In classical approaches to the investigation of study design, in particular sample size, closed form expressions (or reasonable closed form approximations) are used to obtain sample size formulae. The change-point model with unlabelled sub-groups and incomplete follow-up is of such complexity that even reasonable closed form expressions are unavailable. An alternative is to investigate the model using computational methods, and with the modern availability of computing power, it is feasible to conduct a simulation study to investigate classification-error.^27^

The essence of study design is to define a set of criteria and optimise the design to achieve the best value of the criteria, typically under some constraints, e.g. cost and time. For example, randomised control trials are designed to detect a difference between treatments, while minimising the number of patients. We consider Bayesian study design, as we have elected to work in a Bayesian framework, but it is very similar in spirit to frequentist study design.

We define our design criteria to be the precision of parameter estimates, the power to detect a change-point and the classification-error. We may directly affect our criteria by altering the sample size, however as previously discussed, the naive first wave sample size is a poor metric, since we fail to account for attrition. Hence, we consider our expected post-change-point sample size as a combined feature, where the designer can determine a range of possible attrition rates and cohort sizes.

The final design aspects concern the form of the underlying trajectories and the measurements themselves. The measurement error is of fundamental importance as we would expect in sample size calculations, and is typically inherent to the outcome. In our two-class model, change and no change, the key feature of the trajectories is the shift at the change-point, which we term the change-magnitude. It follows that larger change-magnitudes would be easier to detect; however, the parameter that ultimately determines the separation between the classes is the magnitude of the measurement error relative to the change-magnitude. Hence, we consider a range of measurement errors to inform designs with differing measurement variability and also differing change-magnitude ratios.

### 1.5 Outline

In this paper, we perform a simulation study to investigate classification-error, and the power to detect a change-point, in a class of Bayesian (multi-path) change-point models with unlabelled sub-groups. This family of change-point models are commonly used to investigate change^18,28^ though this is a restrictive model (fixed effect), we have extended it to incorporate unlabelled individuals (for whom the sub-group to which they belong is unknown).

The focus of our paper is on the classification-error properties of the study design; within the model, we investigate there are many features to explore. For this paper, we consider two common design parameters: measurement error and attrition. Although we perform a simulation study, our generated data are inspired by the study of cognitive decline as measured by the Mini-Mental State Examination (MMSE),^29^ where it is recognised that not every individual experiences a change and it is of interest to infer the change or no change label. Our setting allows us to gain insight into the issues of classification-error under sample size scenarios with incomplete follow-up and present guidelines for future study designs.

## 2 Methods

When reporting a simulation study, it is important to be clear on the aims, computation details and summary measures.^30,31^ First, we formally define the class of change-point model and the incomplete follow-up mechanism of interest. Next, the details of the Markov chain Monte Carlo (MCMC) method used for Bayesian inference are presented.

Our investigation is motivated by the study of cognitive decline in ageing; using this setting, we define the parameter ranges considered in our simulation study.

Finally, we discuss the issues of sample size determination in relation to our Bayesian approach, specifically the summary measures and statistical criteria for which a sample size is optimal.

The MCMC algorithm was implemented in custom written C code and run in parallel on several multi-core machines to obtain posteriors efficiently. All other analyses were performed using the GNU R statistical software.^32,33^

### 2.1 Change-point model

We consider the class of change-point models commonly known as broken-stick models with fixed effects. The underlying shape consists of a linear trend before and after the change point with potentially differing slopes such that there is no discontinuity at the change-point.

Of note, we extend the model such that each individual is a member of one sub-group with differing slopes around (i.e. before or after) the change-point. In this paper, we consider the case where each individual either experiences a change or not, i.e. there are two distinct sub-groups within the population with one group experiencing no change in the slope.

Individuals may have varying numbers of observations at varying times. Formally, let there be *n* individuals each with *m_i_* observations of the outcome *y_ij_* at time *t_ij_*, for i=1,…,n and $j=1,\ldots ,m_{i}$. For each individual, *r_i_* indicates the group label, i.e. whether they experienced a change (*r_i_* = 1) or not (*r_i_* = 0).

For our simulation study, we consider the case of each individual’s observations being aligned such that $t_{i1}=0\forall i$. Further, the time of the change-point is fixed for all individuals for whom a change occurs, at time *c* say.

**Figure 1.**
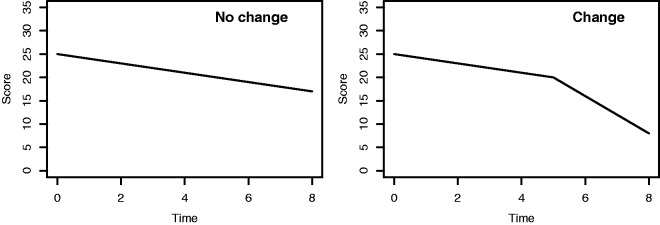
Example illustration of the two distinct sub-groups: no change and change. The two linear segments meet at the change-point with no discontinuity.

Hence, our change-point model includes a mixture of two classes, the so-called no change class (also known as the stable class) and change class, with fixed underlying shape, see Figure 1 for an illustration.

Using a fixed effects model, let *α* and *β* denote the intercept and slope respectively of the linear trend in the no-change sub-group. Define *δ* as the change-magnitude, i.e. the change in the slope after the change-point. The change-point is at some fixed time, *c*, for all individuals whom experience a change. Finally, the observation-error for each individual at each time point is denoted *ε_ij_*. Hence, the general form of this class of change-point models is

(1) $$y_{\mathrm{ij}}=\{\begin{array}{cc} \alpha +\beta t_{\mathrm{ij}}+\varepsilon_{\mathrm{ij}} & \text{ if }r_{i}=0,\text{ or if }r_{i}=1\text{ and }t_{\mathrm{ij}}\leq c\\ \alpha '+(\beta +\delta )t_{\mathrm{ij}}+\varepsilon_{\mathrm{ij}} & \text{ if }r_{i}=1\text{ and }t_{\mathrm{ij}}>c \end{array}$$

To ensure there is no discontinuity in the trajectory, the intercept of the change sub-group (*r* = 1) is set to α'=(α-δc). The requirement to have no discontinuity at the change-point is a feature of the broken-stick class of models, not of change-point models in general.^34,35^

The model specification is completed by defining the probability of an individual experiencing a change, $\text{P}(r_{i}=1)=p_{r_{i}}$, and the observation-error, *ε_ij_*. The observation-error is assumed to follow a normal distribution with precision *τ* ($\tau =\frac{1}{\sigma^{2}}$), and all error terms, both between and within individuals, are independent.

### 2.2 Observation model

Cohort attrition is a significant problem in longitudinal studies, particularly if the aim is to detect the existence and impact of change-points. Hence, we incorporate incomplete follow-up into our simulation study.

When performing a simulation study, the data generating model must be fully specified.^30,31^ Hence, we must specify the time points at which observations are made and a drop-out mechanism for the incomplete follow-up; our so-called observation model, or equivalently a missingness model.

We consider an observational model with a minimally interesting drop-out mechanism to investigate classification-error, monotone random attrition. Individuals are observed at fixed time points, such that $t_{i1}=t_{1}\forall i$, and there is a single drop-out time, $t_{d_{i}}$ with an associated drop-out probability for each individual, $p_{d_{i}}$.

In this paper, we keep the observation model constant across individuals, namely $t_{d_{i}}=t_{d}\forall i$ and $p_{d_{i}}=p_{d}\forall i$, as the missing profile, although of great interest and importance in longitudinal modelling, is not the focus of this study. By focusing on a basic missing profile, we can more easily deduce and present the impact of a varying amount of missing data on classification-error. Thus, each individual *i* is observed at times $t_{1},t_{2},\ldots ,t_{d}$, when they may drop-out with probability *p_d_* and are not observed further, or continue to be observed at times $t_{d+1},\ldots ,t_{m}$.

This pattern of drop-out, where individuals do not return to the study at later time points, is common in cohort studies.

Under this model, drop-out is independent of sub-group, the missingness is so-called MCAR.^22^ The unlabelled change-point model presented is not inherently limited to the MCAR setting. However, to focus on the novel unlabelled sub-groups aspect of the model, we use an MCAR profile in this study. Further work is needed to investigate the characteristics of our model within the more complex, and realistic, settings of MAR and MNAR.^22^

### 2.3 Bayesian inference using MCMC

Within the Bayesian framework, we use Bayes rule to formulate the posterior of interest as the product of the likelihood of the observed data and the prior densities of the parameters. We observe $y=(y_{11},\ldots ,y_{{\mathrm{nm}}_{n}})$ and $t=(t_{11},\ldots ,t_{{\mathrm{nm}}_{n}})$ with a likelihood that is a combination of our fixed effect change-point model and observation model

$$\pi (\alpha ,\beta ,\delta ,\tau |y,t,c,t_{d},p_{d},p_{r})\propto \text{L}(y,p_{d}|\alpha ,\beta ,\delta ,\tau ,t,c,t_{d},p_{r})\pi (\alpha ,\beta ,\delta ,\tau )$$

where in general an individual’s probability of drop-out, $p_{d_{i}}$, may depend on *y_i_*.

The likelihood is complicated by the observation model and unknown sub-group labels for each individual. In fact, for a general observation (i.e. missingness) model with unknown sub-groups the likelihood becomes intractable, meaning that we cannot evaluate the likelihood of a set of observations directly. It would require integrating over many possible missing data values, made more difficult as there are no closed form integrals or conjugate priors.

The likelihood is intractable due to the unlabelled sub-groups. To evaluate it, we need to integrate over both the change and no-change possible scenarios for each individual. Under a Bayesian approach, it is conceptually easy to add change-indicators, $r=(r_{1},\ldots ,r_{n})$, as further parameters by augmenting the parameter space (also known as data augmentation or auxiliary variables).^36,37^ Conditional on the change-indicators, the likelihood is then easily computed for each observation. Hence the posterior of interest is, in the most general terms

$$\pi (\alpha ,\beta ,\delta ,\tau ,r|y,t,c,t_{d},p_{d},p_{r})\propto \text{L}(y,p_{d},r|\alpha ,\beta ,\delta ,\tau ,t,c,t_{d},p_{r})\pi (\alpha ,\beta ,\delta ,\tau ,r)$$

where the likelihood, L(·), is defined by the change-point model and observation model. Using data augmentation, we can now make inference using MCMC methods.

Within our simulation study, we are assuming that drop-out is independent of sub-group, the so-called MCAR setting; hence, *y* is independent of *p_d_*. Further, combined with a constant drop-out probability, $p_{d_{i}}=p_{d}\forall i$, the observation model can be factored out of the likelihood as a constant. Thus, it has no effect on the likelihood and can be ignored; its only effect is to vary the amount of missing data, which will impact the precision of estimates. In our notation

$$\begin{array}{c} \text{L}(y,p_{d},r|\cdot )=\text{L}(y,r|\cdot )\text{L}(p_{d}|\cdot )=\text{L}(y,r|\cdot )CC\in \mathbb{R} \end{array}$$

Finally, the likelihood term involving the sub-group and outcome is separable, as the sub-group labels are augmenting the parameter space to make the likelihood tractable. Leading to the posterior

(2) $$\pi (\alpha ,\beta ,\delta ,\tau ,r|y,t,c,t_{d},p_{d},p_{r})\propto \text{L}(y|\alpha ,\beta ,\delta ,\tau ,r,t,c)\text{L}(r|p_{r})\pi (\alpha ,\beta ,\delta ,\tau ,r)$$

where the first part of the likelihood is given by equation (1).

The mixture of two sub-groups and change-point leads to a non-standard form of the likelihood (i.e. no conjugate prior), and hence the requirement to use Metropolis-Hastings (MH) updates within an MCMC scheme. The proposal distributions within each MH update were of standard forms. However, there is an interesting aspect to the dispersion of the proposals that we will return to in Section 2.4.

The MCMC chains were run for 10^5^ iterations and, as is standard practice, an initial block of 10^3^ iterations were discarded as burn-in. Further, only every 50th iteration was retained, so-called thinning, to reduce the auto-correlation of the approximately 3000 remaining samples from the posterior density.

### 2.4 Simulation study

The family of change-point models, combined with an observation model, as defined in Sections 2.1 and 2.2, have many parameters to consider. Within the scope of this paper, it would not be feasible to consider the full parameter space, due to limits of space and clarity in presenting our results.

If we consider a common study design question, determining a sufficient sample size to reliably detect a pre-defined effect size, a key consideration is the measurement error; more noisy observations require a larger sample size. In our setting, with unlabelled sub-groups, noisy measurements are an obvious feature to investigate. The magnitude of the pre-defined effect size is important, but mainly its magnitude relative to the measurement error.

As already discussed, incomplete follow-up is an important feature of longitudinal study design. Hence, we should consider a parameter from the observation model within our simulation study; in our case, the only parameter is the drop-out probability. As will be discussed in Section 3, we present our results in terms of a sample size summary measure, the expected post-change-point sample size, which reduces the complexity in presenting our results.

There remain several other parameters within equation (2), which we can broadly group into three categories as the focus for future work: observation, shape, and sub-group. Parameters concerning the observation model (i.e. the missingness model), such as the number and timing of observations, and drop-out mechanisms, lead into future investigations of more interesting dependent missing mechanisms (i.e. MAR and MNAR). Parameters concerning the shape of the process, namely the slope, intercept, change-magnitude and change-point, are important for translating the results to other settings, but they are inherently linked to the observation-error; thus, we feel that for our first investigation the observation-error is sufficient. As an intuitive comparison, consider a common approximate approach to determining the sample size for a two sample t-test comparing two population means which only requires the ratio of the variance and effect size^38^; hence, the relative magnitudes of the shape parameters and observation-error are of key importance. Finally, parameters concerning the sub-group labels, which determine the relative numbers of individuals in each group, are set to generate equally likely sub-group membership in this paper, which will likely correspond to a best case scenario for classification.

Thus, we consider two features of our extended change-point model: the effect of the observation-error, *τ*, that is noise or measurement error and the drop-out parameter, *p_d_*. We investigate the interaction of varying *τ* − *p_d_* over different first wave sample sizes in a simulation study.

For a simulation study, we must generate many simulated data sets at a range of parameter values. Rather than considering abstract scales for the parameters, we use a motivating real-world application, modelling cognitive decline in ageing as measured by the MMSE.^29,39^ Change-point models have previously been applied in the field of ageing and cognition,^40^ and for the MMSE in particular^41^ which we use as a basis for our parameter ranges.

The MMSE is measured on a scale from 0 to 30, with scores greater than 25 considered normal cognition. Although the MMSE is discrete, and our focus is on a continuous outcome, assuming discrete outcomes as continuous is common and we primarily use the MMSE to motivate otherwise arbitrary parameter values. As all our individuals are aligned, such that $t_{i1}=0\forall i$, and we set our intercept as 25, *α* = 25, which is mild cognitive impairment on the MMSE scale. However, for our motivating example of 75 year olds, this corresponds to the mean observed MMSE value. Equating a unit of time to one year, when modelling decline in cognition a decline in MMSE score by one per year is reasonable, so the slope of the no change group (before the change-point) is minus one, β=-1. Given the typical decline of one point per annum, a reasonable accelerated rate of decline would be three points per annum, and so to give a slope past the change-point of three would require a change-magnitude of two, δ=-2.

In keeping with our motivating example of studies of cognition in ageing and a yearly time scale, typical studies last five to 10 years with three to five observations (or waves). Thus, we assume five observations at fixed time points for all individuals. Namely, $t_{1}=0,t_{2}=2,t_{3}=4$, $t_{4}=6$, and $t_{5}=8$. We set the time of the drop-out as *t_d_* = 4 and the time of the change-point as *c* = 5 for all individuals. Thus, individuals who drop-out do not have any observations after the change-point. The probability of experiencing a change is the same for all individuals, namely $p_{r_{i}}=p_{r}=0.5\forall i$.

In summary, the parameter values derived from our motivating example, modelling cognitive decline in ageing, are (α,β,δ)=(25,-1,-2) and $\left(c,t_{d},p_{r})=(5,4,0.5\right)$.

Within our simulation study, it only remains to specify the range of the observation-error and drop-out parameters. We consider three error-precisions, τ∈{0.05,0.1,0.2} (error-variances $\sigma^{2}\in \{20,10,5\}$, respectively) corresponding to three very different error magnitudes relative to the observations and a wide range of drop-out probabilities, $p_{d}\in \{0.1,0.3,0.5,0.7,0.9\}$. In longitudinal ageing studies, drop-out rates of 50% are not unknown.

For each of the 15 possible scenarios, three observation-errors and five drop-out probabilities, we generated 150 data sets consisting of 500 individuals. Each individual experiences a change or not, and either has complete information or drops out.

To assess the impact of sample size, we restrict the number of individuals used from each simulated data set. The subsets were defined by restricting to the first *k* individuals in each data set, k=25,50,75,100,125,150,200,300,500. The range of sample sizes was chosen to reflect typical applications of change-point models in the literature.^9,18,40,41^

Having defined the parameter values from our motivating example, it is possible to discuss the scales of the prior and proposal distributions within our Bayesian analysis. The priors are all uninformative and proper

$$\begin{array}{c} \alpha ∼\text{Norm}(0,10^{2})\\ \beta ∼\text{Norm}(0,10^{2})\\ \delta ∼\text{Half}-\text{Norm}(0,10^{2})\\ \tau =(\frac{1}{\sigma^{2}})∼\text{Gamma}(1,1)\\ r_{i}∼\text{Bernoulli}(0.5) \end{array}$$

We use a half-normal prior on the change-magnitude, *δ*, to aid identifiability and, in the case of cognitive decline, the direction of change is known a priori.

The proposal distributions require a slight adaption due to our focus on sample size. With larger sample sizes, the posterior variance is expected to be smaller; hence, using the same proposal distribution across all simulated data sets will induce different mixing and acceptance rates, potentially distorting the comparison across sample sizes. To minimise this, the variance of each proposal distribution was scaled based on the number of observed individuals. Thus, the proposal distributions were

$$\begin{array}{c} q(\alpha '|\alpha )\;∼\text{Norm}(\alpha ,(f(n)1.2{)}^{2})\\ q(\beta '|\beta )\;∼\text{Norm}(\beta ,(f(n)0.85{)}^{2})\\ q(\delta '|\delta )\;∼\delta \times \text{Log}-\text{Norm}(0,(f(n)1{)}^{2})\\ q(\tau '|\tau )\;∼\text{Norm}(\tau ,(f(n)0.85{)}^{2}) \end{array}$$

where $f(n)=\frac{\text{log}(25)}{\text{log}(n)}$, since the minimum sample size considered in our simulation study is 25 (any function such that f:ℕ→(0,1], f(N)→0 as n→∞ and *f* decays to zero at a suitable rate would be appropriate). These distributions and function, *f*, were based on multiple trial runs to investigate acceptance rates and mixing properties of the posterior samples.

In a real application, it may be beneficial to use an adaptive update scheme to improve the efficiency of the MCMC chain. In our simulation study, this deterministic adaption was sufficient.

### 2.5 Bayesian sample size

Frequentist sample size calculations have a long history, particularly in medical research due to clinical trials, and are typically framed in terms of hypotheses with Type I and Type II errors. The null hypothesis, significance level, alternative hypothesis, and desired effect-size combined with optimality criteria define the required sample size.

Bayesian sample size is slightly different and is split into two main types: model comparison using Bayes Factors, which may be extended to a fully decision theoretic approach; or inferential approaches (see Adcock^42^ and Pezeshk^43^ for a review).

The fully decision theoretic approach^44,45^ is the preferred method, accounting for the prior distribution and incorporating a loss-function to quantify the cost of a decision. However, to avoid further complicating the results, and how to translate them to different settings, we discount the use of utility functions as they are very application specific.

The Bayes Factor approach^46^ compares the marginal posterior probability of the data, *D*, under two models, which can be taken as equivalent to the null and alternative hypotheses in the frequentist approach. The Bayes Factor comparing two models is defined as the ratio of the marginal posterior of the data under each model, $\text{BF}=\frac{\pi (D|M_{1})}{\pi (D|M_{0})}$, i.e. the parameters and their priors have all been integrated out (hence the Bayes Factor depends on the prior^47^). Values of the Bayes Factor substantially different from one indicate evidence in favour of one model. A complete simulation study, incorporating varying priors that could be used to make general statements about study design for change-point models would be very difficult to perform and summarise. Since our Bayesian change-point model has no closed form expressions and missing data, the MCMC analysis is computationally expensive for each simulation. Further, it is non-trivial to compute the Bayes Factor from the MCMC output.^48^ Hence, given the scope of our study, we discount investigating varying priors and also the Bayes Factor approach.

The remaining Bayesian approaches are termed inferential,^43^ for example the average length criterion (ALC) and posterior moments. The ALC is defined as the average length of the *ν*% highest posterior density (HPD) interval. The HPD interval is a form of Bayesian credible interval, which is often compared to frequentist confidence intervals (although a confidence interval and a credible interval are two distinct concepts and typically would not coincide). Sample size is then defined in terms of a minimum desired ALC. Thus, the ALC is a sample design criterion, akin to setting the desired significance level and power in a frequentist design setting. Simpler than the ALC, sample size can be defined in terms of a minimum desired posterior moment, such as median, mean or variance.

In our investigation, for the intercept (*α*), slope (*β*), change-magnitude (*δ*) and error-precision (*τ*), we consider the error in the estimated posterior median, the variance of the posterior and the 95% ALC. For the change-indicators (*r*), we analyse the classification-error.

### 2.6 Simulation summaries

For each scenario, we generated 150 data sets, each generating a posterior sample using the MCMC scheme in Section 2.3.

For the intercept (*α*), slope (*β*), change-magnitude (*δ*) and error-precision (*τ*), we compute the posterior median from each data set within a scenario, *s*, and summarise all data sets using the MAE

(3) MAEs(ω)=1150∑l=1150|ω^l-ω*(s)| for ω∈{α,β,δ,τ}and scenarios.

where ω^l denotes the median of the MCMC posterior for parameter *ω* in the lth data set and $\omega^{*}(s)$ its true value (varies by scenario only for *τ*).

Again, for the intercept (*α*), slope (*β*), change-magnitude (*δ*) and error-precision (*τ*), we compute the posterior variance and 95% ALC and summarise all data sets using the mean of the variance or ALC

$${\text{var}}_{s}(\omega )=\frac{1}{150}\sum_{l=1}^{150}\text{var}(\omega_{l}){\text{ALC}}_{s}(\omega )=\frac{1}{150}\sum_{l=1}^{150}\text{ALC}(\omega_{l})$$

We note that the overall summaries are based on 150 data sets. Hence in addition to the error due to using a finite sample from the MCMC posterior, there will be further Monte Carlo error due to using a sample of all possible data sets.^49^ The decision to use 150 repetitions of each scenario is in line with other simulation studies and gives sufficient insight into the relationship of interest.

Within our change-point model, there are two distinct sub-groups, change and no change. This naturally leads to two questions: whether there is a change at all, namely whether δ≠0; and given there is a change-point, what is the classification-error of individuals.

In the classical sample size framework, the Type II error, or power, of the test is the ability to detect a difference when one exists. In the Bayesian framework, we could perform model selection comparing the base *δ* = 0 against δ≠0 (or δ<0 if only modelling decline). Alternatively, we could monitor the marginal posterior probability on *δ*. In the one-sided case it is not sufficient to monitor the HPD interval of *δ* without a spike-and-slab prior^50,51^ (since *δ* is continuous, then P(δ=0)=0). However, the probability P(δ<h) for a range of *h* can easily be computed from the MCMC samples, giving an indication of the probability of a meaningful change-magnitude.

Finally, we summarise the classification-error for each scenario. In our simulation study, the true *r_i_* is known for each individual and we can obtain a posterior probability for whether they experienced a change or not. We plot receiver operating characteristic (ROC) curves as the true positive rate against the false positive rate ranging over all thresholds. In this instance, the posterior of each *r_i_* can be summarised as the probability of being assigned to the change class; we then vary the threshold of assignment to the change class from zero to one. To summarise ROC curves, it is common to compute the area under the curve as a measure of classification-error, our area under the ROC (AUROC). By definition, an AUROC of one corresponds to a perfect classifier and an AUROC of half is random assignment.

## 3 Results

We first illustrate several simulated data sets, to present the qualitative nature of our scenarios and to highlight the noisy properties of the simulated data. Despite having the true generating parameters, we do not expect to recover them perfectly nor do we expect to be able to avoid classification-error.

Within our simulation study, we ran 20,250 MCMC chains covering 150 repetitions of all scenarios: three error-precisions, five drop-out probabilities and nine samples sizes. Given the number of MCMC chains, we considered summary measures of the acceptance rates and convergence; both were sufficient for valid inference under our implementation (see Appendix for further details).

Our aim is to gain insight into the relationship of sample size and classification-error, focusing on the error-precision and drop-out probability. Even moderate tabulated output would be far too verbose, thus we present graphical representations of our results that more succinctly illustrate our findings.

In the following sections, we plot the summary measures defined in Section 2.6 by error-precision against the expected sample size post-change-point. The plots show the mean for each scenario, omitting the uncertainty due to Monte Carlo error for clarity, and LOWESS^52,53^ curves to highlight the trend across the scenarios.

Using these summary plots, we assess change-point detection and classification-error under varying post-change-point sample sizes. Although not our main focus, we also consider the three shape parameters: intercept (*α*), slope (*β*), change-magnitude (*δ*); and inference on the error-precision (*τ*) itself.

### 3.1 Example data sets

Our change-point model was coded in the R statistical software package^32^ and for each scenario – defined by $\tau^{*}$ and $p_{d}^{*}$, together with the values of the other parameters and observation times, see Section 2.4 – we generated 500 individuals’ trajectories; this was repeated 150 times (all data sets available from the corresponding author).

**Figure 2.**
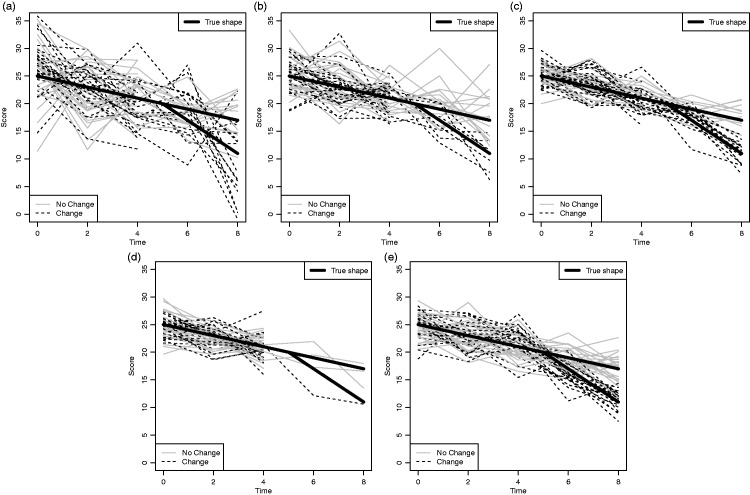
Example data sets under different scenarios with *n* = 75 and common intercept (*α* = 25), slope (β=-1), change-magnitude (δ=-2) and change probability ($p_{r}=0.5$). The thick solid black lines denote the underlying model, and each individual’s observations are drawn depending on whether they truly experience a change (dashed black line) or not (solid grey line). Plots (a)–(c) illustrate differing error-precisions, *τ*, with drop-out probability $p_{d}=0.5$. Plots (d)–(e) illustrate differing drop-out probabilities, *p_d_*, with error-precision τ=0.2.

Figure 2 illustrates five example data sets with *n* = 75 individuals and common intercept (*α* = 25), slope (β=-1), change-magnitude (δ=-2) and change probability ($p_{r}=0.5$).

In our model, each individual is a member of the change or no change class. Figure 2 shows that some individual’s observations are at odds with their sub-group due to observation-error. For example, in Figure 2(b), there are individuals whom did not experience a change (solid grey line) but have similar final observations to those who did experience a change (dashed black line), and vice versa.

### 3.2 Existence of a change-point

To consider the question of classification-error, we must first determine whether distinct sub-groups exist. There are two degenerate cases, in the first a change-point exists and all individuals experience a change. The other degenerate case is when no change-point exists, i.e. the change-magnitude is zero, and all individuals experience no change.

The case of no change-point presents an identifiability issue for our model. If the change-magnitude is truly zero, then the change and no change sub-groups follow identical shapes; meaning the labels are ill-defined. Conversely, the case of all individuals experiencing a change given a non-zero change-magnitude is well defined, since the labels correspond to distinct shapes.

Hence, before we can consider classification-error, we must first address the existence of a change-point, i.e. check for a non-zero change-magnitude. As discussed in Section 2.5, we could model the existence of a change-point and, using Bayes Factors or variable selection approaches, obtain appropriate posteriors. However, for the reasons discussed earlier, we shall not directly compare the evidence for a change-point but instead consider the posterior probability of the change-magnitude being non-zero. Recall, by definition P(δ<0)=1 and P(δ=0)=0 (continuous distribution restricted to the negative real line), so instead we set some threshold, *h* < 0, and consider P(δ<h). The choice of *h* must not be too small or it will give no information; based on the relative parameter values and motivating example, we let *h* = 0.05.

**Figure 3.**
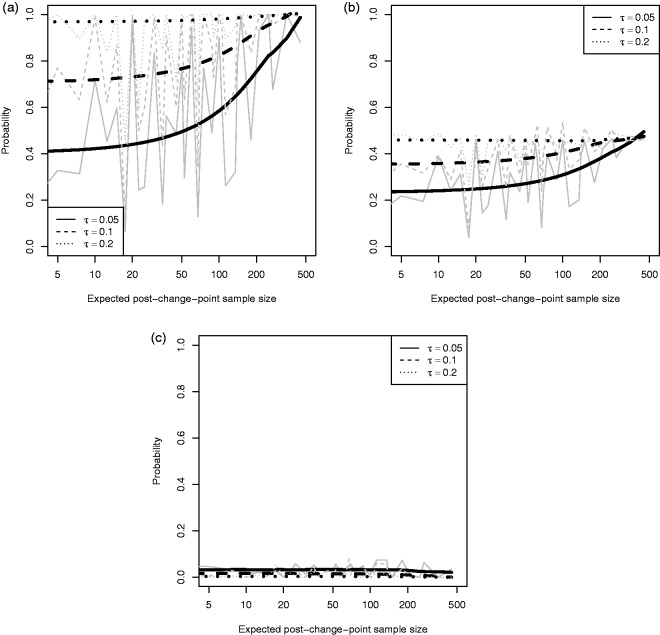
Mean posterior probability of change-magnitude, *δ*, being less than (a) –0.05, (b) –2, and (c) –3; given the true change magnitude is $\delta_{\text{true}}=-2$. We use P(δ<-0.05) as an indicator that a change-point exists. Scenarios are grouped by the true observation-error (solid, dashed and dotted lines). Simulation summaries are grey lines and LOWESS curves are black.

Figure 3 plots P(δ<h) for our detection threshold, h=-0.05, and two further values: the true value of the change-magnitude, *h* = – 2, and a value below the truth, *h* = – 3.

Figure 3(a) reflects our explanation of failing to detect a change-point. We see for the smallest error-precision scenario, τ=0.05, that for post-change-point sample sizes less than 100 there is (averaged across our 150 repetitions) less than half of the posterior mass of the change-magnitude parameter away from zero. Crudely speaking, we only have a 50% chance of detecting a true change-point. This increases to 70% at twice the precision, τ=0.1, and approximately 95% at four times the precision, τ=0.2. For all error-precisions, we see that a post-change-point sample size of 500 almost guarantees detection of a non-zero change-magnitude.

Note that Figure 3(a) only indicates that the posterior density is away from zero, not that it is centred around the true parameter. It is perhaps concerning that even for large post-change-point sample sizes, say 200 individuals, under our reasonable error-precision (τ=0.1), there is still a 10% chance of failing to estimate a non-zero change-magnitude; and hence failing to properly classify any individuals.

Assuming reliable inference, we would typically expect the posterior to be approximately symmetric about the true parameter value ($\delta_{\text{true}}=-2$). Figure 3(b) indicates that this is the case, as the post-change-point sample size tends to larger values. That is, in the limit across all observation-error values, half the posterior probability is below the true value and half above, i.e. $\text{P}(\delta <\delta_{\text{true}})\to \frac{1}{2}$.

Finally, to illustrate the directionality of the inference, Figure 3(c) plots the posterior probability of a change-magnitude smaller than –3. Note that the order of the three error-precision lines is reversed on this plot compared to Figure 3(b), as we are considering the opposite tail of the posterior; namely the solid line corresponding to τ=0.05 shows the lowest probabilities in Figure 3(b), but the highest in Figure 3(c). For the smallest error-precision (τ=0.05), there is a non-zero probability of a change-magnitude greater than –3, indicating that we can both under and over estimate this parameter while still being bounded at zero.

### 3.3 Classification error

Classification is inference at the level of individuals, unlike inference on the change-point model parameters which is at the population level (in a fixed effects model). Classification-error concerns differences between an individual’s true label and their inferred label. However, this comparison is only well defined, if both the true and inferred labels are interpretable. That is, unless we first confirm the existence of a change-point the inferred labels, change and no change, are meaningless. Hence, change-point detection is more fundamental to our problem.

We first consider the issues of ill-defined labels. Then, once we have determined the existence of a change-point, following Section 3.2, meaning that our change and no change labels are well defined, we can consider the accuracy of classification.

**Figure 4.**
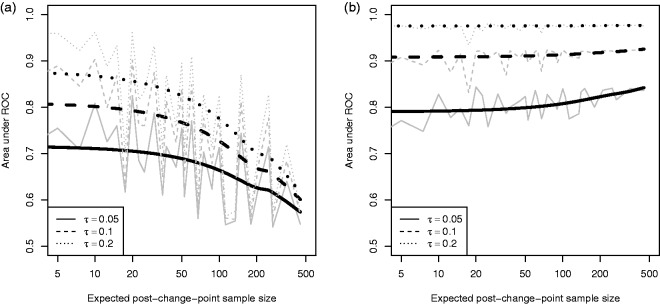
Area under ROC curve for (a) all individuals and (b) only those individuals with observations after the change-point. The plot for all individuals seems to indicate larger sample sizes lead to a loss in accuracy, but this is an unfair comparison due to the inclusion of individuals who drop-out before the change-point – their classification is ill defined. Hence, the second plot considers only individuals with observations past the change-point.

Figure 4(a) plots the mean AUROC by error-precision over a range of post-change-point sample sizes. Counter to our expectation, the classification-error becomes greater for larger sample sizes and appears to be tending to an AUROC of 0.5, i.e. random labelling. We do not expect classification to become worse with an increasing sample size.

Recalling our simulation setting from Section 2.4, individuals who drop-out are lost to follow-up before the change-point, so they cannot really be in either the change or no change group. This issue arises since we are considering inference at the individual level, unlike in Section 3.2, where at the population level the question of the existence of a change-point is well defined.

In fact, we might consider these individuals to be in a distinct third group, for whom the question of whether they experience, a change-point is ill defined. Such a group is distinct since individuals are MCAR, and the drop-out probability is independent of sub-group. Hence, there is no information in the observed data (scores *y* and times *t*) to inform the sub-group label for individuals with incomplete follow-up; they will have random labels. Thus, for these individuals, the AUROC will approach a half.

Typically at larger post-change-point sample sizes, there are more individuals with incomplete follow-up, leading to a large number of random labels. These random ‘true’ labels will lead to many mis-classification errors from our inference, since both the ‘true’ label and posterior label will be random the probability they coincide is dependent on the proportion of change and no change individuals in the population.

For our setting, with known fixed change-point location and MCAR drop-out, we can exactly define three distinct sub-groups: change, no change and dropped-out before change-point. Figure 4(b) plots the classification-error for the subset of individuals with observations past the change-point, that is individuals for whom classification into change or no change is well defined. Hence, we have removed the effect of including the dropped-out individuals. Immediately, we see that classification improves with sample size, as we expect.

When interpreting Figure 4, we must account for detecting a change-point as indicated by Figure 3. Until a change-point is reliably (we deliberately leave the term reliably undefined) detected, the AUROC appears steady, then begins to climb slowly. Even at the largest post-change-point sample sizes, we do not achieve perfect classification. Further, there is an indication that for increasing sample size the AUROC is levelling out to some limiting value. This is in keeping with our earlier observations on Figure 2 that there exist individuals with observed outcomes that are closer to the opposite sub-group, namely some individuals in the no change group have outcome scores – due to the observation-error – close to the change group. Hence, we do not expect to recover perfect classification.

We must also consider that for a post-change-point sample size of 10, combined with an equal chance of experiencing a change or not ($p_{r}=0.5$), we expect only five individuals to be in each sub-group. However, this is not fixed to be exactly five, and due to the random group assignment several of the simulated data sets had no individuals in one of the sub-groups. Thus, at low post-change-point sample sizes, there will be higher Monte Carlo error (recall that the group membership of individuals was not passed to the MCMC analysis code, so the inference is made blinded to the true group membership).

At moderate post-change-point sample sizes, the probability of detecting a change-point increases. However, the simulation study summaries of the Bayesian posteriors for the change-magnitude (*δ*) will reflect this uncertainty by becoming a mixture of two distributions: no change with posterior mass around zero and change with posterior mass around two. Hence, Figure 3 is really summarising a bimodal distribution. Bimodal distributions are poorly characterised by the variance and ALC (the ALC being ill defined when the posterior is multimodal), which we shall consider in Sections 3.5 and 3.6, so it is important to consider all results in the context of the existence of a change-point.

### 3.4 Post-change-point sample size

In study design, the often asked question is the required sample size. This is well defined in single time point studies, for example the classic comparison of means between two groups. However, in the longitudinal setting, we have the additional complexity of attrition.

The problem of attrition is especially troubling in a change-point model; in the worst case scenario, we might lose all individuals before any experience the change-point. There are some parallels with designing studies to investigate time-to-event processes, requiring long enough follow-up to capture sufficient events to make inference.

With that background in mind, we proposed a convenient metric to encapsulate both the first wave sample size (at time *t*_1_) and attrition; our so-called expected post-change-point sample size. This metric has the benefit of focusing study designers thinking on the key aspect of the change-point process, while still being a univariate summary of the sample size.

For our cognitive decline inspired process defined in Section 2.4, we have two observation times beyond the change-point (*c* = 5) and drop-out time (*t_d_* = 4); both of which are fixed for all individuals. Thus, for a given sample size, *n*, and drop-out probability, *p_d_*, there is an expected number of individuals who should be observed at the last observation ($t_{5}=8$), namely $n(1-p_{d})$.

There are two important observations about our metric. Firstly, it is possible to achieve the sample expected post-change-point sample size with different first wave sample sizes. For example, *n* = 100 coupled with $p_{d}=0.1$, and *n* = 300 coupled with $p_{d}=0.7$, both result in an expected post-change-point sample size of 90. Secondly, the actual post-change-point sample size is random. This means our smooth curves – for example in Figure 3 – are really smoothing variability in the mean absolute error, over the y-axis, and across the actual post-change-point sample size from each simulated scenario, over the x-axis.

**Figure 5.**
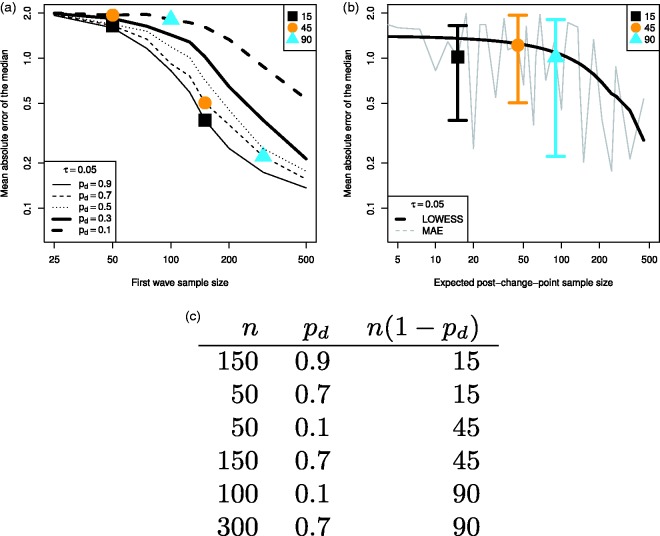
Mean absolute error of the posterior median estimate of the change-magnitude, *δ*, plotted against (a) the first wave sample size and (b) our expected post-change-point sample size. We highlight three expected post-change-point sample sizes: 15, 45 and 90, and the (c) the corresponding first wave size, *n*, and drop-out, *p_d_*, paris.

In Figure 5 we illustrate the benefit of considering our results in terms of the expected post-change-point sample size using the bias in estimating the change-magnitude parameter, which we shall consider in more details in Section 3.5. The change-magnitude parameter, *δ*, is reasonably “well behaved” and clearly dependent on the number of observations post-change-point.

Figure 5(a) presents the mean absolute error for the change-magnitude (*δ*) parameter when τ=0.05, plotting the x-axis as the first wave sample size, with five different lines for each drop-out probability, *p_d_*. We have highlighted three expected post-change-point sample sizes: 15, 45 and 90, which can each be obtained as two distinct combinations of *n* and *p_d_*. In Figure 5(b), we plot the same information with the x-axis as our expected post-change-point sample size. The ‘error bars’ on each expected post-change-point sample size reflect the mean absolute errors from Figure 5(a).

The general pattern, larger sample size leads to less bias, is clear across both plots. However, the interaction of attrition and first wave sample size in Figure 5(a) is, even for the “well behaved” change-magnitude, complex and highly non-linear. Conversely, Figure 5(b) presents a noiser picture in the raw data, but a clearer understanding of the impact of observations after the change-point on our ability to make inference. This is particularly important more complex questions of change-point detection in Section 3.2 and classification-error in Section 3.3, where our message is that post-change-point sample size is a key consideration. However, we acknowledge, as clearly shown in Figure 5(b), that our simple metric exhibits variability. Hence, our use of LOWESS curves highlights the trends of interest.

### 3.5 Intercept, slope and change-magnitude

**Figure 6.**
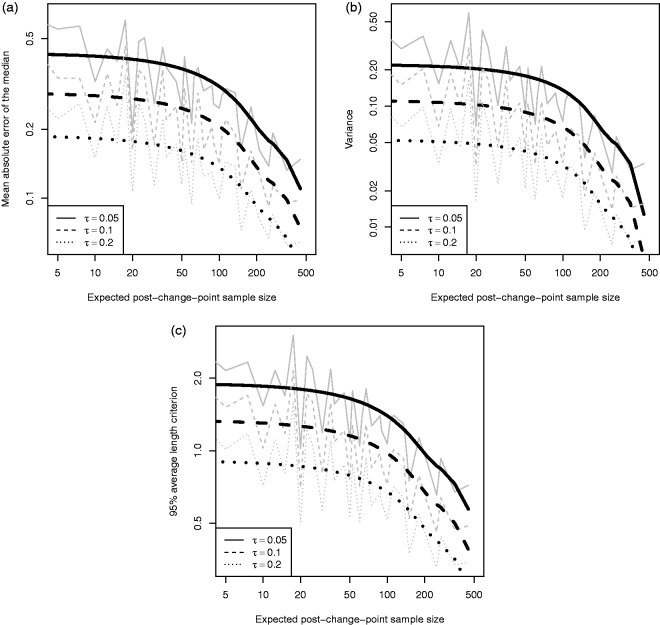
Summary measures for the intercept (*α*) parameter over a range of post-change-point sample sizes: (a) mean absolute error of the posterior median, (b) mean posterior variance and (c) 95% ALC. Scenarios are grouped by the true observation-error (solid, dashed and dotted lines). Simulation summaries are grey lines and LOWESS curves are black.

**Figure 7.**
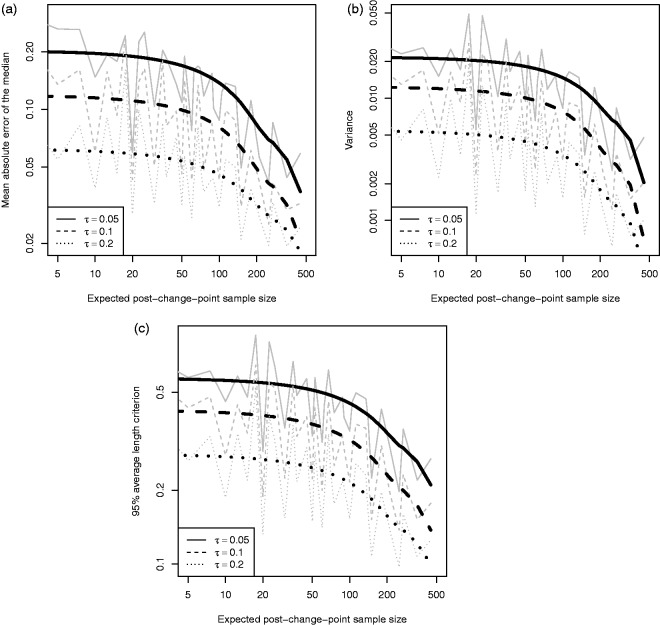
Summary measures for the slope (*β*) parameter over a range of post-change-point sample sizes: (a) mean absolute error of the posterior median, (b) mean posterior variance and (c) 95% ALC. Scenarios are grouped by the true observation-error (solid, dashed and dotted lines). Simulation summaries are grey lines and LOWESS curves are black.

**Figure 8.**
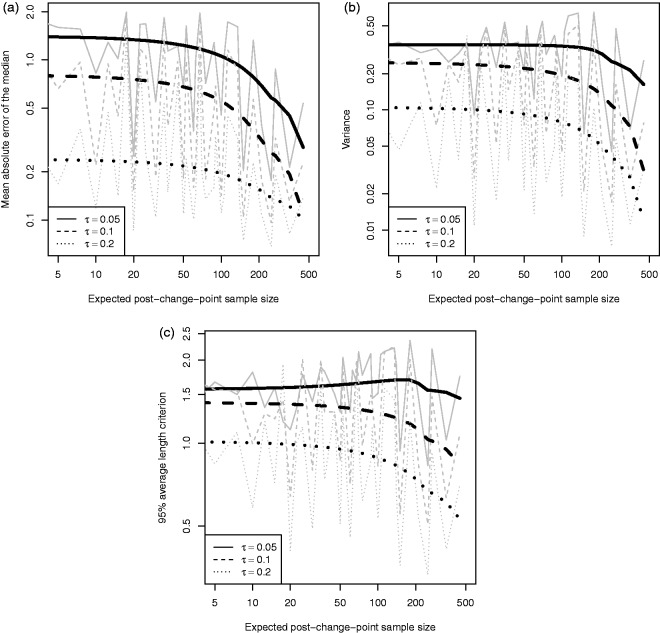
Summary measures for the change-magnitude (*δ*) parameter over a range of post-change-point sample sizes: (a) mean absolute error of the posterior median, (b) mean posterior variance and (c) 95% ALC. Scenarios are grouped by the true observation-error (solid, dashed and dotted lines). Simulation summaries are grey lines and LOWESS curves are black.

Our change-point model, defined in equation (1), has three parameters that determine the underlying shape: the intercept (*α*), slope (*β*) and change-magnitude (*δ*), see Figure 1. Figures 6 to 8 plot: (a) the mean absolute error of the posterior median, (b) the mean posterior variance, and (c) the 95% ALC against the expected post-change-point sample size, namely $n(1-p_{d})$. The scenarios are separated based on the true observation-error, which takes one of three values (τ∈{0.05,0.1,0.2}).

By definition the shape parameters are dependent, since we require continuity at the change-point. Beyond the continuity constraint, a deeper dependence is induced by the existence of a change-point. If the change-magnitude is zero or, almost equivalently, all individuals belong to the no change class, then the slope parameter will be informed by all observations. Conversely, if a change-point does exist, then the variation is explained by both the slope and change parameters. These two situations will result in different estimates for the slope, which in turn will affect the estimate for the intercept.

Figure 6(a) plots the bias in the posterior median for the intercept (*α*) parameter. We see that for small post-change-point sample sizes, there seems to be a plateau. Beyond a post-change-point sample size of 50, on the log-log scale, the bias seems to decrease linearly. Given drop-out is MAR, we would expect the bias in the intercept to be well behaved. Figure 6(b) and (c) is closely related, for a unimodal symmetric posterior there is a one-to-one relationship between the variance and ALC. As expected, the measures seem well behaved on the log–log scale for increasing post-change-point sample size.

Similarly, Figure 7 plots the same measures for the slope (*β*) parameter and the behaviour is comparable.

The change-magnitude (*δ*) parameter behaves very differently. In Figure 8, the bias is an order of magnitude greater than for the slope parameter despite being comparable, and the variance and ALC do not appear as well behaved.

The explanation of Figure 8 comes from the ability to detect the existence of a change-point. If there is insufficient evidence of a change-point then the posterior for the change-magnitude will be close to zero (in a classical sense or using spike-and-slab priors, P(δ=0)≈1). In this case, recalling that the bias is bounded in one direction since we assumed a half-normal prior, the mean absolute error must be equal to two. Thus, at small post-change-point sample sizes, when we cannot reliably detect the existence of a change-point we see a large bias. However, once we reliably detect the change-point, the bias reduces rapidly, as seen in Figure 8(a). This also explains the plateau on Figures 6(a) and 7(a), at these sample sizes, the posterior detects no change-point and so the intercept and slope are being fitted to the entire data (akin to a simple linear regression).

### 3.6 Error-precision

The error-precision (*τ*) parameter is particularly important to our discussion. Typically in classical sample size formulae, the user must specify the observation-error a priori as a fundamental aspect of determining the sample size. In that regard, we consider the error-precision as known. In an application, we would also wish to make inference on the error-precision.

**Figure 9.**
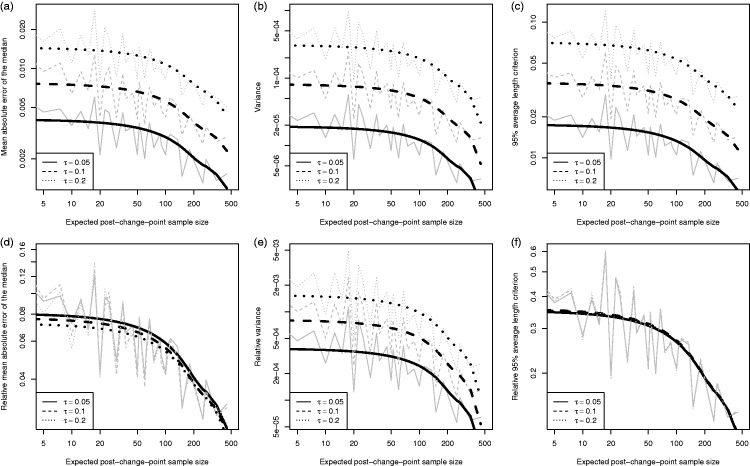
Summary measures for the error-precision (*τ*) parameter over a range of post-change-point sample sizes: (a) mean absolute error of the posterior median, (b) mean posterior variance and (c) 95% ALC. Scenarios are grouped by the true observation-error (solid, dashed and dotted lines). Simulation summaries are grey lines and LOWESS curves are black.

Figure 9(a) to (c) plots the summary measures for the error-precision as for the shape parameters in Section 3.5. Against our intuition, the least precise scenario, τ=0.05, (equivalently, the noisiest observation-error, $\sigma^{2}=20$) has the lowest bias, variance, and ALC.

However, this comparison is unfair as the measures are not based on the same values across the three scenario groups; unlike the plots in Section 3.5, where the scenario groups were all comparing their measures to the same true value. To compensate, Figures 9(d) to (f) plots the bias, variance and ALC relative (by scaling) to the true error-precision.

Once scaled, ignoring the low post-change-point sample sizes due to the failure to detect a change-point, we see that there is no difference in the bias and ALC, both of which appear to decrease linearly on the log–log scale. This is as expected since all observation errors are independent, so we would expect fairly reliable inference about the error-precision.

## 4 Discussion

Using a simulation study, for a class of fixed effect change-point models with unlabelled individuals belonging to either a change or no change class, we have investigated the relationship between post-change-point sample size and classification-error – as well as the bias and variance of the posterior estimates of the fixed effects – in a Bayesian framework.

### 4.1 Insights into study design

The results relate to our motivating setting of modelling decline in cognition measured by the MMSE. Although exact numerical results would need adjusting under different parameters, the overall trends and conclusions are widely applicable.

Our first insight is to consider study design in terms of (expected) post-change-point sample size, a function of the drop-out probability (*p_d_*) and first wave sample size (*n*). We have shown important relationships that hold for a range of sample sizes and missingness scenarios by reducing these two factors to a single number.

The price we pay for using a simple univariate sample size metric is greater variability across comparable scenarios, where we define comparable scenarios to be *n* − *p_d_* pairs giving similar post-change-point sample sizes ($n(1-p_{d})$). However, the between comparable scenario variability must be considered within the context of the within scenario Monte Carlo variability. Recall that each scenario has 150 simulated data sets, each with a random post-change-point sample size (distributed around the expected post-change-point sample size). The within and between scenario variability is of a similar magnitude, meaning that combining comparable scenarios as illustrated in Section 3.4 is reasonable.

Study designers can consider the trade-off between the cost to decrease drop out (thus minimising incomplete follow-up) and the cost of initial recruitment to obtain a specified post-change-point sample size. Further, thinking in terms of post-change-point, sample size highlights the issue of biased attrition in cohort studies,^20^ since only a sub-sample of initial recruits are represented post-change-point. Our drop-out model is appropriate for unbiased attrition processes and we leave the impact of alternative attrition models for future work.

Our main result combines change-point detection and classification-error. As expected, the error-precision defines the limiting AUROC for larger post-change-point sample sizes. That is, given the separation of the true underlying sub-groups relative to the error-precision, we would expect an upper limit on the classification accuracy, i.e. for very low error-precision the two sub-groups would overlap and be indistinguishable. The observation-error is a key aspect of required sample size, and the effect of larger precision (equivalently, smaller variance) can be trivially seen in Section 3. In our study, an error-precision of 0.05 (equivalently a standard deviation of 4.47) has a limiting AUROC of approximately 0.85. A more realistic error-precision of 0.1 (standard deviation 3.16) for the MMSE^29^ has a limiting AUROC of approximately 0.9.

For study designers, the inherent limit on classification is a concern. Worse, the rate of increase to the limit is very slow. Thus, only minor improvements in AUROC are gained for substantial increases in post-change-point sample size.

Conversely, and unintuitively, for low post-change-sample sizes, there is not a significant increase in classification-error (equivalently a decrease in the AUROC). However, the stability in classification-error is an artefact of the half-normal prior on the change-magnitude. Since the parameter can never be zero, even a slight change-magnitude results in a difference in the likelihood between the two labels for an individual. Thus, individuals who are far above or below the true shape will be ‘correctly’ labelled. Despite these ‘correct’ labels, the labels themselves cannot be interpreted (and are essentially meaningless), since if the change-magnitude is zero then both sub-groups really experience no change.

Thus, when designing a study optimised for classification, we require a post-change-point sample size that gives interpretable labels, i.e. confirms the existence of a change-point, and then attains the desired classification-error. Hence, reliability of change-point detection is more important to setting sample size than the classification-error. From Figure 3 we see that, in our motivating example, a post-change-point sample size of 50 is the minimum at which the probability to detect a change-point increases across all three error-precisions.

### 4.2 Comparison to real applications

The novel aspect of our change-point model is to include unlabelled sub-groups, which has allowed us to gain insight into the issue of classification-error; many change-point models assume every individual experiences a change or pre-classify the individuals. Within our simulation study, we considered individuals to be equally likely to be in either group; this is the most optimistic setting to detect a difference with equally size groups. However, we did not include any covariates that could aid classification, which may reduce the sample size but would add further coefficients to estimate – a trade-off that requires further investigation.

The inclusion of unlabelled sub-groups allows us to consider links between our approach and methods to test for the existence of a change-point; specifically, if all individuals are classified in the no-change group, we have evidence that there might not be a change-point at all. Ji et al.^19^ consider a hypothesis test for the existence of a change-point using MMSE scores of 47 individuals (a subset of a larger dataset), where some exhibit so-called accelerated decline (i.e. a change-point) under visual inspection. The individuals’ observation times are zero aligned at diagnosis of Alzheimer’s Disease, so in our framework there is a post-change-point sample size of 47. According to our design criteria, this sample size has only moderate power to detect a change-point. Further, our approach would accommodate a mixture of change and no-change individuals, whereas the hypothesis test of Ji et al.^19^ only considers everyone to have a change-point or not.

Although we have used a simplified setting, this class of fixed effect change-point models have been applied in practice.^18,28^ Hall et al.^18^ study the Buschke selective reminding test and, in our notation, estimate an intercept (*α*) of 46.06, slope (*β*) of –0.61, a change-magnitude (*δ*) of 1.49, and an approximate observation-error (*τ*) of 0.4, which reasonably match our simulation study values derived from the MMSE. With a post-change-point sample size of 365, according to our design criteria, their study was appropriately powered to detect a change-point. However, as part of their approach Hall et al*.*^18^ also estimate the time of the change-point. In terms of our sample size results, the effect of also estimating the change-point would be to under-estimate the power.

### 4.3 Summary

In our simulation setting, the sub-group label is inferred solely on the observed scores and times. In real application, focusing on classification other covariates, such as gender, would likely be available. Previous work has considered adding covariates in equation (1) under a Bayesian^54^ or frequentist^55^ framework. The effect of adding covariates into the change probability model – which was held constant in this paper, $p_{r_{i}}=0.5\forall i$ – as a logistic model with covariates has not been considered and remains an open question for further research.

The expected post-change-point sample size metric focuses designers thinking on the key aspect of study design for change-point processes, while still being a relatively interpretable univariate summary of the required sample size. It may also be possible to extend this univariate metric to more complex attrition models, beyond the single drop-out time model.

We have shown that even for studies of modest size (*n* = 500, with 50 past the expected change-point) in the fixed effect analysis a change-point of size two can be detected and modelled. Further work is needed to extend these results to more complicated change-point models and to assess the relationship of sample size with the change-magnitude and the observation model. We have developed initial guidance for study designers on the relationship between accuracy of classification and sample size.

## Appendix. Checking MCMC mixing and convergence

Our simulation study involved running 20,250 MCMC chains (three error precisions and five drop out probabilities, on each of the 150 data sets at nine different sample sizes). Each run typically took between 30–60 min, and overall computation time was a week on three 48-core servers (48 × 2.2 GHz cores with shared memory running Ubuntu linux).

Mixing and convergence are important for the validity of the posteriors but it would be infeasible to manually assess the mixing and convergence of each chain. Due to the computational burden of the simulation study, it was not possible to run multiple chains on each data set. Hence, standard automated checks comparing between and within chain variance^56^ were not available. A random sample of 20 chains, across all scenarios, was manually assessed for convergence by inspecting trace plots and auto-correlations (results not shown). Acceptance rates within each chain for all parameter updates were recorded, and overall summaries were plotted. Despite using adaptive proposal distributions, see Section 2.4, the acceptance probability of all updates still decreased with sample size. However, the acceptance rate was sufficient given the number of iterations and choice of thinning.

Every chain was initialised with the same starting point, namely $\left(\alpha_{0},\beta_{0},\delta_{0},\tau_{0})=(20,3,-10,1\right)$, as an unlikely point in the parameter space that was far from the truth. This choice enabled us to assess convergence by monitoring the burn-in periods. For a sample of simulated data sets, alternative initialisation points were used to test the convergence from multiple start points. In all cases, the chains from the separate starting points converged to a common posterior within the burn-in period.
